# Pigeons (*C. livia*) Follow Their Head during Turning Flight: Head Stabilization Underlies the Visual Control of Flight

**DOI:** 10.3389/fnins.2017.00655

**Published:** 2017-12-01

**Authors:** Ivo G. Ros, Andrew A. Biewener

**Affiliations:** ^1^Department of Organismic and Evolutionary Biology, Harvard University, Cambridge, MA, United States; ^2^Division of Biology and Bioengineering, California Institute of Technology, Pasadena, CA, United States

**Keywords:** head stabilization, turning flight, *Columba livia*, sensory feedback control, gaze

## Abstract

Similar flight control principles operate across insect and vertebrate fliers. These principles indicate that robust solutions have evolved to meet complex behavioral challenges. Following from studies of visual and cervical feedback control of flight in insects, we investigate the role of head stabilization in providing feedback cues for controlling turning flight in pigeons. Based on previous observations that the eyes of pigeons remain at relatively fixed orientations within the head during flight, we test potential sensory control inputs derived from head and body movements during 90° aerial turns. We observe that periods of angular head stabilization alternate with rapid head repositioning movements (head saccades), and confirm that control of head motion is decoupled from aerodynamic and inertial forces acting on the bird's continuously rotating body during turning flapping flight. Visual cues inferred from head saccades correlate with changes in flight trajectory; whereas the magnitude of neck bending predicts angular changes in body position. The control of head motion to stabilize a pigeon's gaze may therefore facilitate extraction of important motion cues, in addition to offering mechanisms for controlling body and wing movements. Strong similarities between the sensory flight control of birds and insects may also inspire novel designs of robust controllers for human-engineered autonomous aerial vehicles.

## Introduction

The ability to maneuver, turn, and maintain stable flight has been critical to the evolutionary diversification and success of flying animals. Such aerial maneuverability requires rapid sensory integration with motor control of the wings, body, and tail. However, the mechanisms by which sensory input is coupled to motor output for maneuvering flight in birds has been understudied compared to studies of avian functional anatomy, neural organization and sensory neurophysiology (for review, see Zeigler and Bischof, 1993).

Sensory input clearly shapes behavior, but behavior can also shape sensory perception (Zeil et al., 2008). For instance, fly flight is characterized by brief sharp turns alternated with periods of straight translational flight (Schilstra and van Hateren, 1998). By confining visual motion induced by self-rotation, or angular optic flow, to these rapid turns, a flying animal's course, speed, and distance information can be more easily extracted from translational optic flow that occurs during straight flight periods (Land, 1999). Such features of flight behavior are, therefore, inferred to improve the quality of sensory perception in flies (Nakayama, 1985; Zeil et al., 2008). Consequently, understanding the relationship between sensory input and behavioral output is a key first step to elucidate behavioral and sensing-related adaptations, as well as how they interact, for robust flight control.

Translational optic flow appears to guide the flight behavior of several unrelated vertebrate and invertebrate species, indicating that it may provide a general visuomotor control stimulus. When flying down a corridor, budgerigars, and honeybees follow flight paths that balance left and right lateral optic flow induced by their translational movement (Srinivasan et al., 1991; Bhagavatula et al., 2011). Additionally, as optic flow increases, budgerigars, bees, moths, fruit flies, and blowflies reduce their flight speed to maintain an optic flow rate below a possible internal limit (David, 1979; Srinivasan et al., 1996; Fry et al., 2009; Verspui and Gray, 2009; Bhagavatula et al., 2011; Kern et al., 2012).

Similarly, by moving their head backwards and forwards relative to the body just before landing (but not after take-off), pigeons may use fluctuations in translational head speed relative to their surroundings to increase close-range perception of a landing site (Green et al., 1994). These in-flight head speed fluctuations are reminiscent of the head bobbing observed during walking in many birds. However, in contrast to the periods of stationary head “hold” phases that occur during walking (Dunlap and Mowrer, 1930; Friedman, 1975; Frost, 1978), a pigeon's head never comes to a complete stop during landing flight. Based on associations of head orientations during turning flight in pigeons and lovebirds (Bilo et al., 1985; Kress et al., 2015), as well as ascending and descending flight in pigeons, and jumping and slope walking in domestic chicks (Davies and Green, 1988; Green, 1998), the control of head movements has been proposed as an essential underlying component of the visual control of avian locomotion.

Similar to insects, vertebrates possess specialized brain regions tuned for processing features of visual information. Birds, in particular, have well-developed brain regions adapted for visual information processing (Iwaniuk and Wylie, 2007). Two of which are parallel visual pathways, the tectofugal and accessory optic systems (AOS), that contain specialized mechanisms to process visual motion (see Simpson, 1984; Frost et al., 1990). The tectofugal system is tuned to small-field object motion, whereas the AOS is particularly responsive to self-induced translational and rotational optic flow (see Burns and Wallman, 1981; Morgan and Frost, 1981; Frost et al., 1990). Two nuclei within the AOS, the nucleus of the basal optic root (nBOR) and the lentiformis mesecephali (LM), encode incoming optic flow with differences in specific feature sensitivity and directionality (Wylie and Frost, 1990).

In addition to optic flow, cervical mechanoreceptors assist flight control in insects, including dragonflies (Mittelstaedt, 1950), flying locusts (Goodman, 1965), and blowflies (Land, 1973; Liske, 1977). Similarly, reflexes related to cervical and vestibular systems in normal and labyrinthectomized hand-held pigeons led to comparing the flight control system of birds to the autopilot of an airplane. From such studies, Groebbels (1926, 1929) proposed that birds control body motion by tracking head motion, essentially “following their turning heads.” In support of this hypothesis, certain wing and tail muscles in the pigeon react to vestibular stimulation, angular visual stimulation, and passive or active lateral head deflections when under simulated flight conditions (Bilo and Bilo, 1978, 1983). Observations of maneuvering pigeons, zebra finches, and lovebirds provide further evidence that head stabilization likely plays a role in flight control (Bilo et al., 1985; Davies and Green, 1988; Warrick et al., 2002; Eckmeier et al., 2008; Kress et al., 2015).

Visual control of bird flight may therefore depend on combined features of head stabilization and movements during flight. Nearly all animals “foveate,” changing their gaze, or viewing direction, in an alternating pattern of stable gaze fixations and fast saccades, defined as rapid movements of the eye (Land, 1999). However, gaze changes in most birds are primarily driven by head (rather than eye) movements (Gioanni, 1988; Gioanni and Sansonetti, 1999; Maurice and Gioanni, 2004), with independent eye movements related to nearby discrimination tasks (Martinoya et al., 1984). Furthermore, the control of eye position within the head has been shown to be under active control in pigeons (Nalbach et al., 1990), likely providing an offset-position that allows for a common reference frame between visual and vestibular systems (Wylie et al., 1998), and for retinal specializations related to head pose (Wallman and Letelier, 1993). Because of this, head orientation is often used as an indicator of center of gaze direction in birds (Gioanni, 1988; Green, 1998; Eckmeier et al., 2008; Kjaersgaard et al., 2008; Kress et al., 2015).

**Figure 1:**
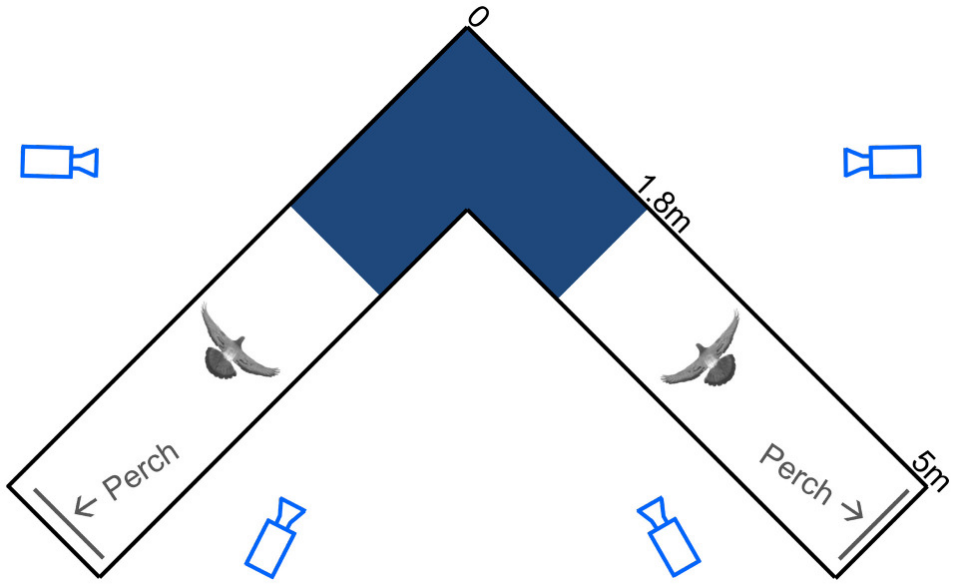
Schematic top view of the flight corridor. Light blue camera outlines represent viewing angles, with camera distances underrepresented by 50%. The spatially calibrated section of the 90° turn (dark blue), the pigeon silhouettes, and the perches (gray lines) are drawn to scale. Dimensions are noted along the outside of one leg of the symmetrical corridor.

**Figure 2:**
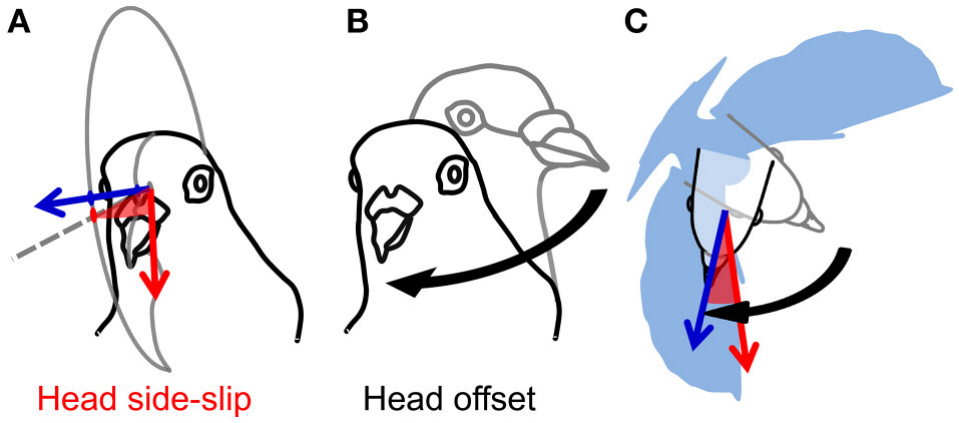
Estimates of visual and cervical proprioceptive feedback. **(A)** Visual feedback is represented by head side-slip (red, shaded wedge); the angle between the head flight velocity, or bearing vector (red arrow), and its projection on the mid-sagittal plane of the head (gray dashed line on gray partial circle, respectively). Head orientation indicates central axis of gaze (blue vector), or viewing direction. **(B)** Cervical proprioceptive feedback is represented by head offset (black curved arrow); the 3D angle between a straight-flight head orientation (gray outline of the head) and the instantaneous head orientation (black outline of the head). Note that **(B)** does not represent a head saccade; the 3D angular difference in orientation of the head is calculated with respect to the straight-flight reference position of the head relative to the body. **(C)** Head side-slip depends on the flight velocity, whereas head offset depends on the relative body orientation (blue silhouette; not to scale).

Following from these studies, we use measurements of head orientation to probe the link between head stabilization and sensory inputs for the control of turning flight in pigeons by analyzing detailed 3D movements of the head and body during low-speed, 90° level turns (Figure 1; Video S1). Measurements of head velocity and orientation provide estimates of visual feedback during turning flight. Assuming negligible eye movement relative to the head (Gioanni, 1988; Gioanni and Sansonetti, 1999), and because a flying animal's self-motion induces whole-field retinal image motion, or optic flow (Gibson, 1958; Koenderink, 1986), deviations between a pigeon's translational movement direction and its head's orientation produce contralateral asymmetries in optic flow during flight. We express these deviations as the bird's “head side-slip”: the angle between the head (flight) velocity and its projection on the mid-sagittal plane of the head (Figure 2A). In addition to this estimate of visual feedback, we infer possible cervical proprioceptive feedback from flexural and twisting movements of the body relative to the head, defined here as “head offset” (Figure 2B). We use these estimates of visual and proprioceptive feedback to examine how each is temporally correlated with subsequent body rotations that redirect aerodynamic force to control turning flight. We use *body rotations* as the output of flight control, given that pigeons change their flight trajectory and orientation through body attitude changes, much like helicopters (Ros et al., 2011) and similar to fruit flies (Fry et al., 2003), during low speed flight.

We hypothesize that head side-slip predicts these body rotations because the most direct measure of the required amount of steering is the deviation between the animal's current and desired flight trajectory. By directing its gaze in the desired flight direction, a bird creates a visual offset between its gaze and the current flight direction proportional to the amount of steering required. We also expect that the amount of neck bending, or head offset, may stimulate afferent cervical proprioceptors to correct changes in body attitude resulting from steering maneuvers. We therefore hypothesize that head offset correlates with subsequent rotation of the body to re-align with the head as the pigeon turns, but not with changes in flight trajectory.

## Materials and methods

### Ethics statement

Three wild-type pigeons (*Columba livia*) were housed and studied at the Concord Field Station (Bedford, MA). This study was carried out in accordance with the recommendations of Harvard University's Institutional Animal Care and Use Committee. The protocol was approved by the Institutional Animal Care and Use Committee.

The pigeons were trained to fly back and forth between two perches situated at either end of two 5-m-long by 1-m-wide by 2-m-high netted sections, connected by a 90° turn midway (Figure 1). The symmetrical, square-corner corridor was constructed of lightweight, 2-cm mesh nylon deer netting supported by a PVC frame consisting of 4-cm diameter piping. No frame elements were positioned on the inside of the turn.

The pigeons were marked at seven anatomical locations, and the bill tip was used as an additional, natural marker. All artificial markers consisted of polystyrene foam and were attached using thermoplastic adhesive. On the head, two 4-mm diameter spheres were attached to feathers near the lateral ends of the coronal suture of the skull. On each wing, at 67% of the length of the ninth primary feathers, 4-mm diameter spheres were attached to the dorsal side of the rachis. On the body, three 19-mm diameter hemi-spheres were attached to 5-cm by 10-cm strips of elastic tape (to reduce feather movement). One marker was placed ventrally over the rostral end of the keel, and two markers were placed on the left and right rump (4-cm lateral to the vertebral column, over the synsacrum).

Using four synchronized high-speed cameras, 3D positions of the markers were reconstructed within a calibrated 2.9 m^3^ volume that encompassed the turn (calibrated corridor section length, width and height through the turn = 2.8, 1.0, and 1.0 m, respectively; Figure 1). Only trials in which the birds did not contact the netting were accepted for processing. All complete wingbeats, from start of downstroke to end of upstroke, captured within the calibrated volume were included for analysis. The synchronized video system, consisting of one FastCam 1024 PCI, and three FastCam SA3 cameras (Photron USA Inc.), recorded at 250 Hz with 0.001 s exposure time. A total of 10,624 video frames were digitized using DLTdv5 (Hedrick, 2008) in Matlab (Mathworks Inc.). Data processing and calculations were also performed in Matlab using custom-written scripts. Positional data were filtered with a fourth-order Butterworth filter using a low-pass cutoff frequency four times the wingbeat frequency. The cutoff frequency was determined by residual analysis (Winter, 2005).

Wingbeats were partitioned into upstroke and downstroke phases, based on reversal of the major bending direction of the primary feathers. This bending reversal of the primary feathers coincided with the instant the primary feather markers moved laterally relative to the body, in both ventral (start of upstroke) and dorsal (start of downstroke) positions.

Visual feedback can be deduced from head velocity and orientation, assuming that the eyes maintain a constant orientation within the head. The head velocity vector relative to the mid-sagittal plane of the head therefore indicates contralateral retinal asymmetries in translational optic flow. Head position was represented by the midpoint between the eyes, and calculated based on the three head markers. Head velocity was determined by the time-derivative of 3D head position.

Gaze indicates where the bird's head is “facing,” defined as the direction of a head-fixed vector in the mid-sagittal head plane and 30° above the line running through the center of the head and the bill-tip (Figure 2A). This gaze vector lies within the plane formed by the lateral canals of the vestibular systems, which are held close to horizontal during level flight (Erichsen et al., 1989; Wallman and Letelier, 1993).

Angular head saccades were identified objectively whenever the angular speed of the head surpassed the propagated positional measuring error for the head markers (0.35 mm) (**Figure 4D**). This measuring error, based on the root mean square deviation between the known size of an object and marker-based measurements throughout the turn, was propagated using the product of the measuring error and the partial derivative of the conversion from marker positions to angular head speed. The temporal measuring error was considered negligible. Even though the absolute spatial measuring error for the larger body markers (similarly estimated at 1 mm) was likely higher than for the smaller head markers, the relative measuring errors were comparable, due to the larger spacing of the body markers.

Saccade duration was measured over the period during which gaze changed outside of the noise level with respect to the phases of head stabilization. However, because saccades started before and stopped after gaze changed outside of the noise level on either side of a saccade, these measurements likely underestimated actual saccade durations. The missing period of gaze change within the noise level at the start and end of saccades was estimated to average 9 ± 2 ms across all saccades. For simplicity this averaged additional duration of the saccades was nevertheless omitted.

Estimates of both visual and cervical proprioceptive feedback were tested as predictors of body rotations. Head side-slip, the angle between the head velocity vector and the mid-sagittal plane of the head, was assumed to represent visual feedback reflecting asymmetries in left vs. right optic flow (Figure 2A). Head offset, the angle between the instantaneous head orientation and a straight-flight reference based on the instantaneous body orientation, was assumed to represent cervical proprioceptive feedback (Figure 2B). Note that head offset changes due to both head saccades and body rotations. Head side-slip and head offset were measured at the end of a saccade, or, for wingbeat cycles without saccades, at the average phase of saccade termination, which occurred 34% into the upstroke (**Figure 5A**). Head side slip was considered positive if gaze was directed into the turn, relative to the flight direction. Head deviation was considered positive if the bird rolled into the turn.

These estimates of sensory feedback were compared to two functionally separate body rotation components, based on previous findings that pigeons produce aerodynamic forces in a uniform direction relative to their body during turns (Ros et al., 2011): (1) *aerodynamic roll/pitch*: body rotations that redirect the aerodynamic force, analogous to helicopter roll and pitch; and (2) *aerodynamic yaw*: body reorientations about the direction of the aerodynamic force, analogous to helicopter yaw (Figure 6). Kinematic data from left turns were mirrored and expressed as right turns. Aerodynamic roll/pitch (ARP) rotations were designated positive if the bird pitched or rolled into the turn. Similarly, aerodynamic yaw (AY) body rotations were considered positive in the direction of the turn.

Analyses were based on a total of 49 wingbeat cycles from nine left and right turns in three individuals. Unless noted otherwise, results are expressed as mean ± *SD*. Correlations were tested with Least Squares Linear Regression models (*N* = 3; JMP, SAS Institute, Cary, NC, USA). These mixed effect statistical models included random effects of bird identity and turn direction. These model results thus correct for apparent correlations that are based on biases in either turn direction or individual differences, and provide single test statistics that apply to trends across all three individuals. To account for multiple comparisons (*n* = 44, the number of statistical tests) a Bonferroni corrected significance level of *p*_c_ < 0.0011 was used to identify statistically significant trends (Shaffer, 1995).

## Results

**Figure 3:**
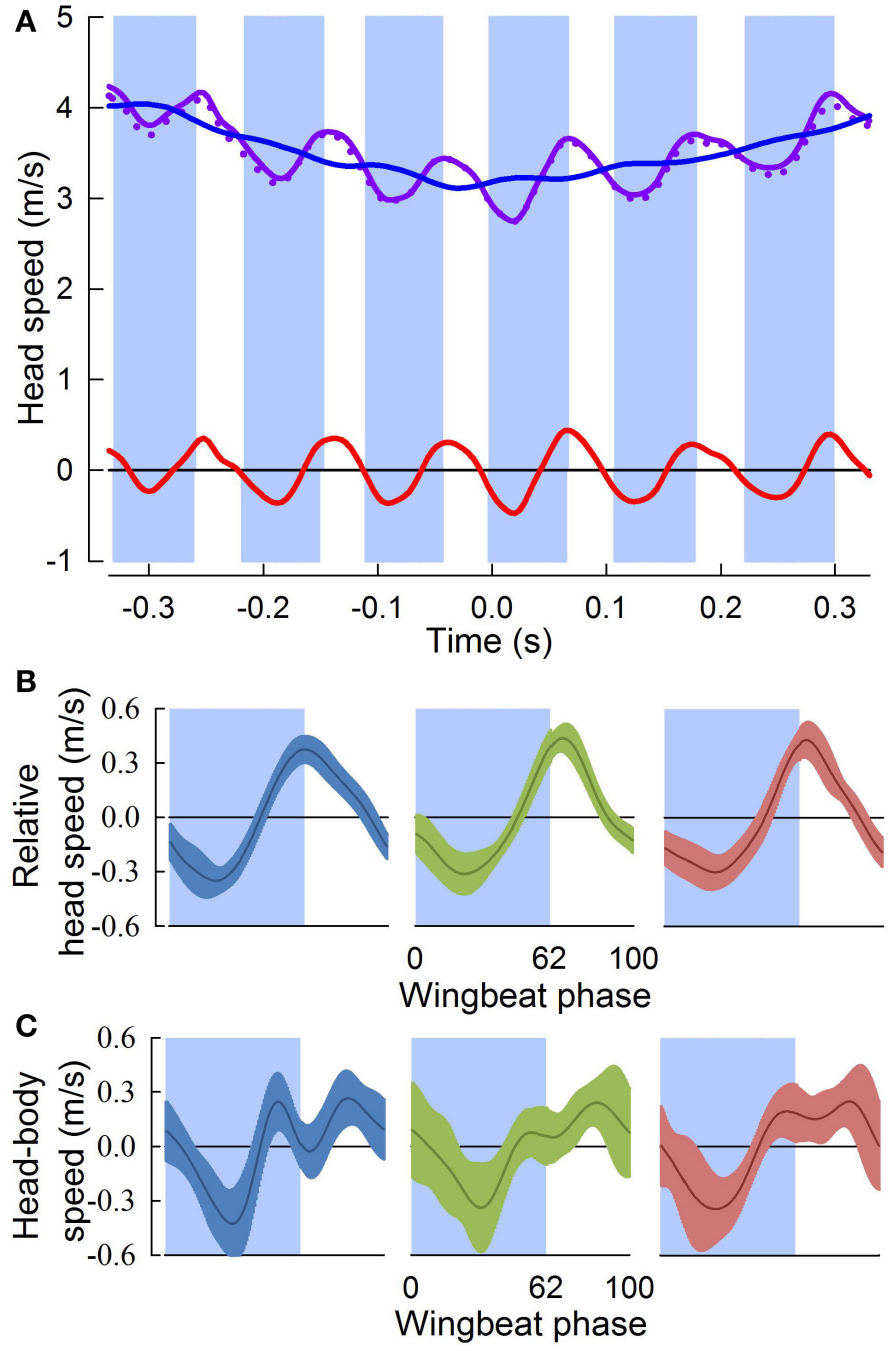
Head speed fluctuates throughout aerial turns. **(A)** Head speed (solid purple trace), and its horizontal component (dotted purple trace), fluctuate throughout a representative turn. Downstroke periods (blue shading) are defined relative to the center of the turn (Time = 0 s); upstroke periods are unshaded. Subtraction of the running-average (blue trace) from the instantaneous head speed gives relative head speed (red trace). **(B)** Relative head speeds (mean ± sd) normalized to wingbeat period for three individuals (blue, *n* = 18; green, *n* = 16; and red, *n* = 16). Head speed peaks just after (4 ± 6 ms) the down-upstroke transition. **(C)** Head speed relative to the body (gray trace) for the same wingbeat cycles in **(B)**. The head does not maintain a fixed distance from the body indicating that active and passive properties of the neck control head motions relative to the body, such that fluctuations in relative head speed **(B)** are not due to oscillatory body motions.

The three pigeons flew through the 90° level turn in 5.5 ± 0.5 wingbeat cycles over a period of 0.63 ± 0.06 s. Perch-to-perch flights lasted 24 ± 1 wingbeat cycles, with 9 ± 1 wingbeat cycles prior to and following the turn. Throughout the turns, the 3D translational speed of the head fluctuated with an amplitude of 0.79 ± 0.14 m/s, with a consistent minimum occurring near mid downstroke, and a maximum 4 ± 6 ms after the down-upstroke transition (Figures 3A,B). Fluctuations in head speed were predominantly horizontal (99.4 ± 0.6%), for head speeds ranging from 2.2 to 4.5 m/s. Head speeds relative to the body, compared to overall head speed fluctuations, similarly showed a minimum near mid downstroke, but were more variable and did not reflect the peak following the down-upstroke transition (Figure 3C).

**Figure 4:**
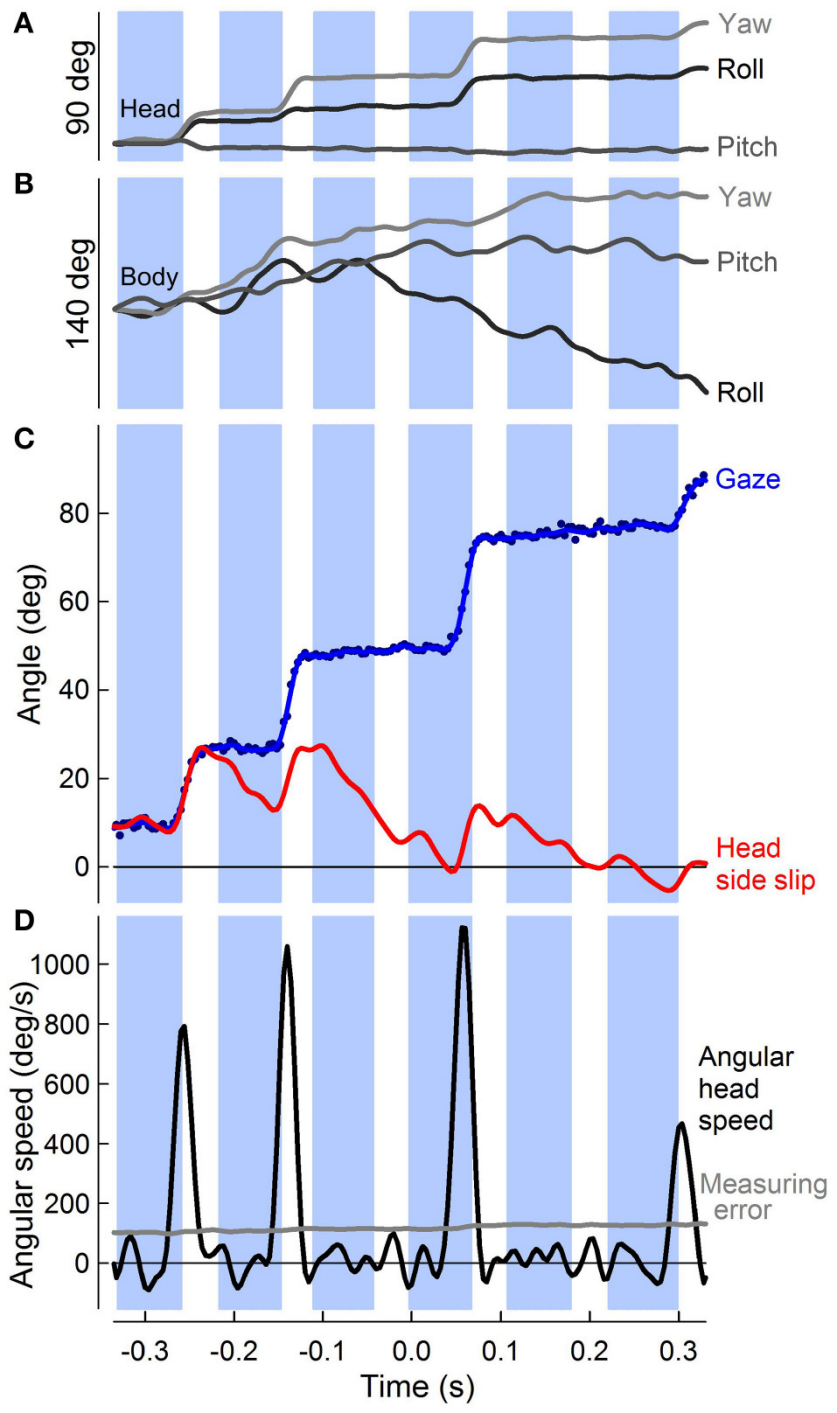
Punctuated head stabilization during aerial turns: PIgeon heads alternate angular stabilization with fast saccades. **(A)** Rotations about the roll, pitch, and yaw axes of the head (dark, medium, and light gray traces, with positive roll, pitch, and yaw defined as right ear down, bill up, and bill right, respectively) over six wingbeats of a 90° turn, during which four “hold” and four saccade phases are observed. Head saccades involve both yaw and roll rotations of the head, with generally little contribution of pitch, reflecting the horizontal flight path of the turn. **(B)** Roll, pitch, and yaw of the body, defined similarly to **(A)**. Body rotations are continuous throughout the turn and distinct from head rotations. **(C)** Unfiltered (blue markers) and low-pass filtered (blue trace) horizontal component of gaze (central axis defined by head direction; see Figure 2A) vs. time during a turn. Gaze changes are directed into the turn (positive). Head side-slip (red trace), defined as the angle between the head velocity, or flight bearing vector, and the mid-sagittal plane of the head peaks following three successive saccades during the turn and one as the bird completes the turn. **(D)** Peaks in horizontal head angular speed (black trace, *n* = 4) that supersede the measuring error (gray trace) indicate angular head saccades. **(A–D)** Downstroke timing (blue shading) is referenced to the center of the turn for this representative trial.

**Figure 5:**
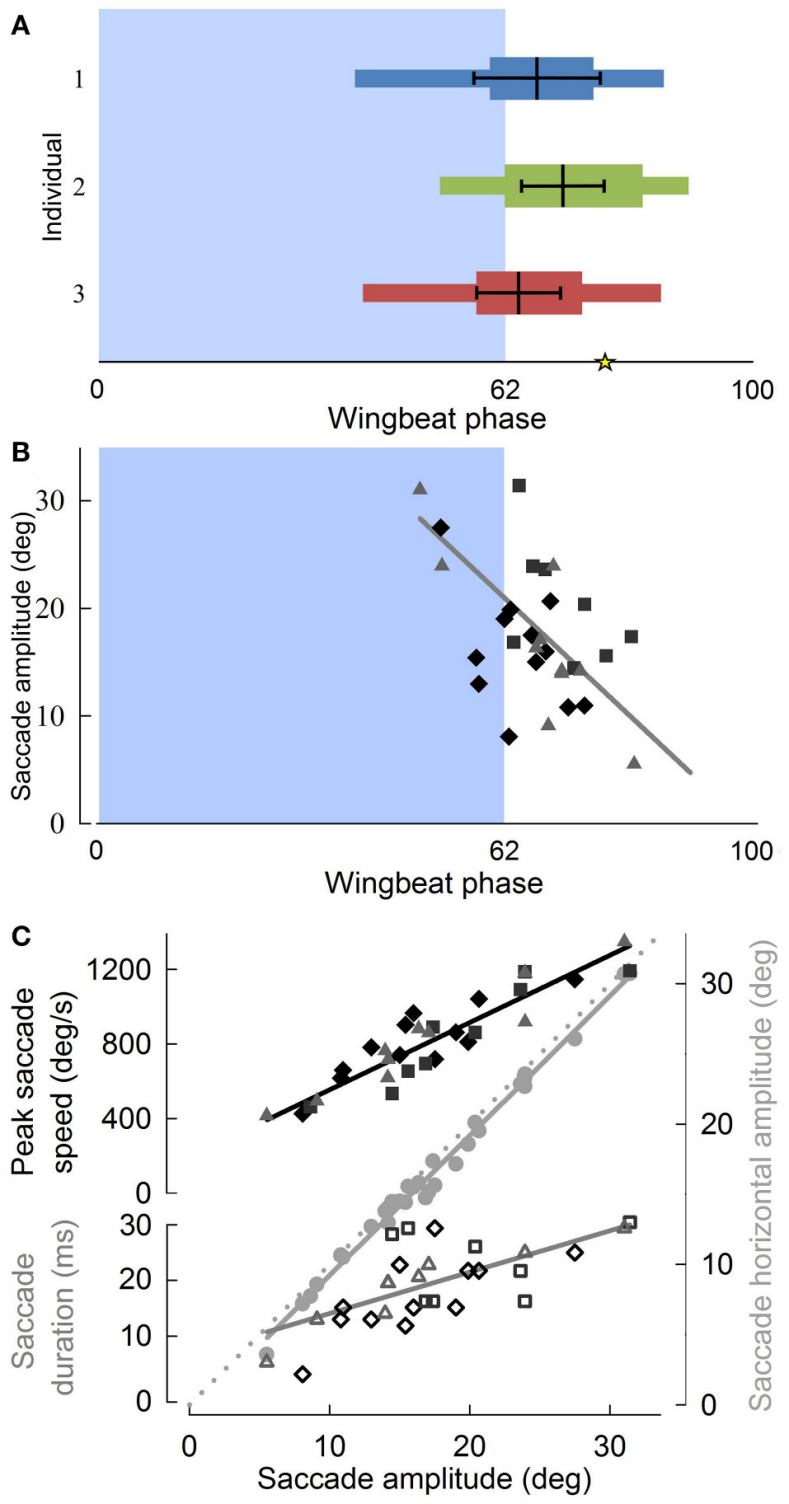
Pigeon angular head saccades occur at the down-upstroke transition during turning flight, are oriented into the turn, and reveal that horizontal saccade amplitude is strongly correlated with saccade duration. **(A)** The timing of angular head saccades normalized to wingbeat duration, for three individuals (blue, green, and red) reveals that head saccades are consistently initiated at the down-to-upstroke transition of the wingbeat cycle. Narrow horizontal colored bars indicate the ranges of saccade start and end phases, whereas wide colored bars indicate average saccade duration, measured from average saccade start to end phases. Thick black lines within the colored bars indicate the mean (vertical black line) ± SD phase of the saccade angular speed peaks. The asterisk indicates the average end phase of observed head saccades. **(B)** Saccade amplitude as a function of wingbeat phase, with symbol and shade indicating different individuals. Larger saccades occur earlier in the wingbeat cycle (*R*^2^ = 0.49). **(C)** Both saccade speed (filled, *R*^2^ = 0.83) and saccade duration (unfilled, *R*^2^ = 0.46) increase with saccade amplitude. Peak saccade speed and duration trend lines indicate their relative contributions to saccade amplitude. Their scaling relative to the proportionality line (dotted gray line, slope = 1) based on constant average duration and speed shows that saccade amplitude is modulated 62% more strongly by speed than by duration. Saccades occur predominantly in the horizontal plane, as indicated by a slope of 0.995 between 3D saccade amplitude and its horizontal component (gray circles, *R*^2^ = 0.99). **(B,C)** Solid lines represent standard least squares regression models, corrected for turning direction and individual effects (*p* < 0.0001 for all four trends; Table S1). Saccade timing metrics were computed with respect to the wingbeats within which they occurred.

Although translational head speed fluctuated continuously, pigeons displayed distinct periods of 3D angular head stabilization, despite continuous rotations of the body (Figures 4A,B). Periods of angular head stabilization were interrupted by brief head repositioning movements, or saccades, lasting 17.6 ± 6.1 % of the wingbeat period and occurring in nearly two-thirds (63 ± 7 %) of the turning wingbeats (Video S2). These angular head saccades were characterized by step-wise changes in horizontal gaze (Figure 4C). All head saccades were directed away from the flight trajectory and into the turn. Peaks in the speed of gaze change occurred near the downstroke-upstroke transition, immediately following peaks in translational head speed (Figures 4D, 5A). Identification of head saccades was based on the speed of horizontal gaze change surpassing the propagated kinematics measuring error (Figure 4D). Saccades varied in magnitude (5–30 deg), duration (4–30 ms), and speed (400–1,200 deg/s). Larger saccades occurred earlier in the wingbeat cycle (*p* < 0.0001; Figure 5B), lasted longer (*p* < 0.0001), and reached higher peak speeds (*p* < 0.0001; Figure 5C; Table S1). Saccade amplitude was modulated 1.62 times more strongly by speed than by duration. Saccades were predominantly horizontal (slope between 3D saccade amplitude and its horizontal component = 0.995, *N* = 3; Figure 5C), consistent with the level nature of the flight turns that were studied.

**Figure 6:**
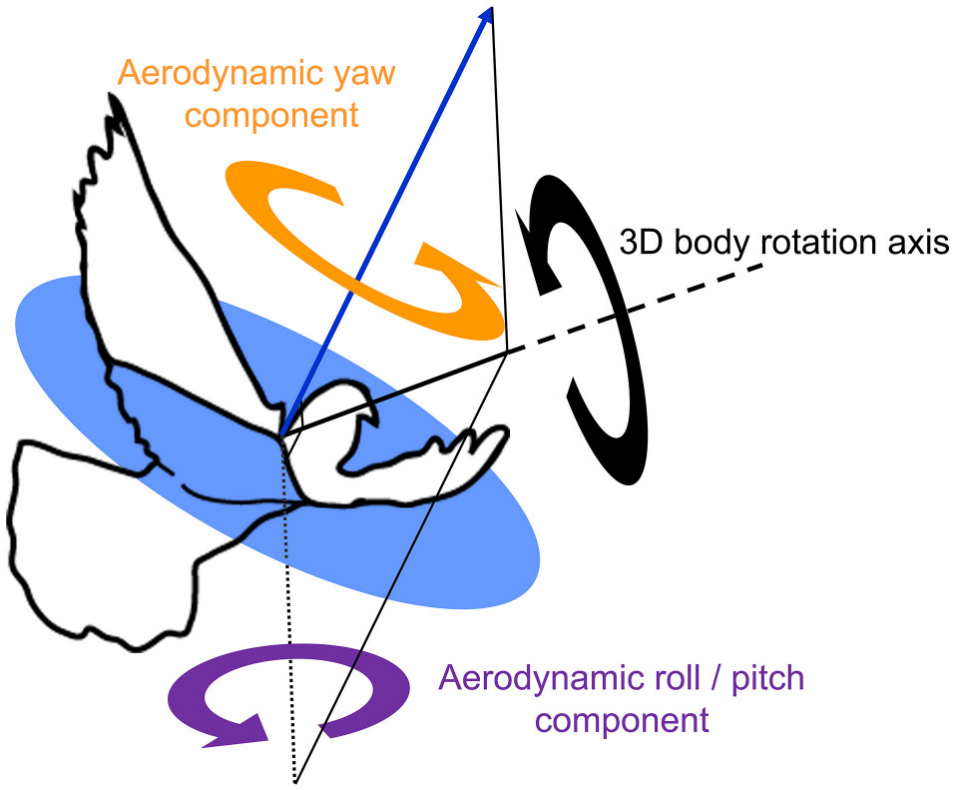
Two functionally separate body rotation components contribute to turning flight. An outline of a pigeon, superimposed with the average direction of aerodynamic force production during the downstroke (blue arrow, after Ros et al., 2011), together with an exemplary 3D body rotation (black circular arrow) about a single axis of rotation (thick black solid line with dashed extension). The 3D rotation is decomposed into two perpendicular components referenced to the net downstroke aerodynamic force: aerodynamic roll/pitch (ARP, purple circular arrow), and aerodynamic yaw (AY, orange circular arrow). Note that the axis describing ARP lies within the circular blue plane, which is normal to the aerodynamic force. ARP is therefore the component of the 3D body rotation that redirects the aerodynamic force; whereas AY represents re-orientation of the body about the aerodynamic force and, therefore, does not cause changes in flight trajectory.

**Figure 7:**
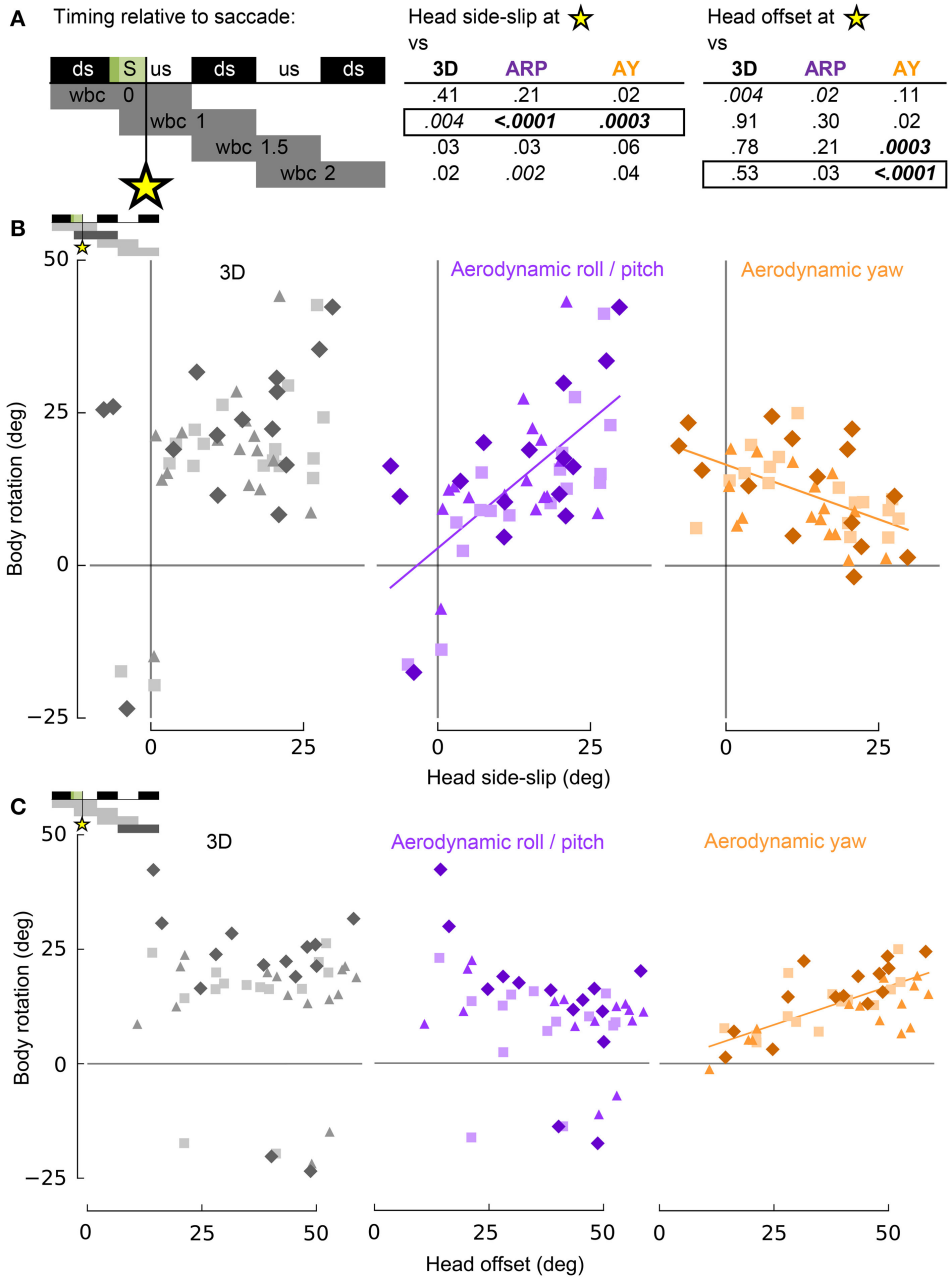
Head side-slip and head-offset induced by saccades provide putative sensory inputs that control subsequent body rotations associated with turning flight. **(A)** Representation of the relative timing between head side-slip and head offset measured at the end of each saccade that occurred during a turn (green area, S), and subsequent body rotations that occurred over successive wingbeat cycles. Downstroke (ds) and upstroke (us) timing is shown in black and white, respectively, for each wingbeat cycle. Gray bars below indicate periods over which body rotations where calculated, relative to each saccade (four rows for four discrete progressive regressions with statistical results reported to the right for head side-slip and head offset). The asterisk indicates the relative timing of the end of the saccades. *P*-values for least squares regression analyses, corrected for individual effects and turning direction, are shown in upper right table for correlations with 3D, aerodynamic roll/pitch (ARP) and aerodynamic yaw (AY) body rotations. Statistical significance was corrected for multiple comparisons (*p*_c_ = 0.0011). Only regressions for boxed-in *p*-values of the upper right table are plotted in **(B,C)**. **(B)** Head side-slip is positively associated with ARP over the wingbeat cycle (wbc 1) immediately following saccades (*R*^2^ = 0.40; *p* = 0.0002) and negatively associated with AY (*R*^2^ = 0.25; *p* = 0.0001). **(C)** Head offset, the 3D degree of neck bending and/or twisting, positively correlates with AY over the second, subsequent relative wingbeat cycle (wbc 2; R^2^ = 0.55; *p* < 0.0001). **(B,C)** Neither head side-slip or head offset exhibits a significant linear correlation with 3D body rotations. Significant regressions are represented by solid purple and orange lines.

**Figure 8:**
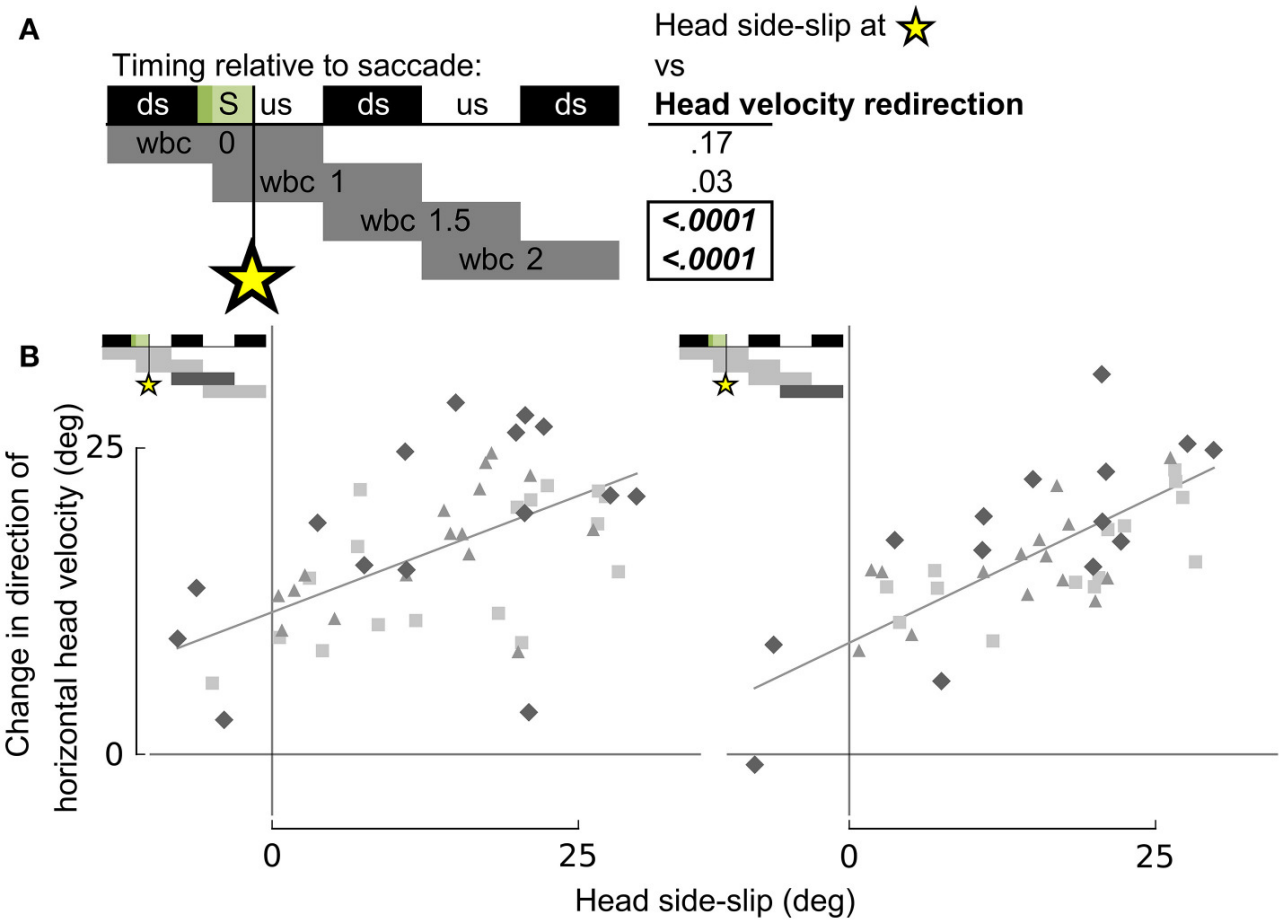
Head side-slip precedes subsequent changes in flight velocity direction. **(A)** Representation of the relative timing between head side-slip at the end of a saccade (green area, S) and changes in direction of horizontal head velocity during subsequent wingbeat cycles (wbc 1.5 & 2). Gray bars below indicate periods over which changes in head velocity direction were calculated relative to each saccade. Regressions for highlighted significant *p*-values in table to the right are plotted in **(B)**. **(B)** Head side-slip is positively associated with changes in horizontal velocity direction over the subsequent 1.5 wingbeat cycles (wbc 1.5 & 2) following a saccade (*R*^2^ = 0.437 and *R*^2^ = 0.51 for the left and right panel, respectively; Table S1).

Estimates of visual (head side-slip) and cervical proprioceptive (head offset) inputs were compared to body rotations during turning flight. These estimates are independent measures with respect to the head, with head side-slip depending on the relative flight direction and head offset depending on the degree of neck bending, and or twisting, resulting from body rotations relative to the stabilized head (Figure 2). Head side-slip and head offset were uncorrelated (*R*^2^ = 0.05; *p* = 0.88). The body rotations, representing behavioral outputs of turning flight, were calculated discretely over whole wingbeat cycles to integrate over finer scale body motions that occur within wingbeats, but which are unrelated to net changes in body orientation (Figure 4B; Warrick, 1998; Hedrick and Biewener, 2007). Correlations of head side-slip and head offset were tested against body rotations and flight velocity changes over four relatively timed wingbeat cycles, with overlapping phases, defined as: (1) wbc 0, which included the preceding downstroke; (2) wbc 1, which included the subsequent downstroke; (3) wbc 1.5, from the subsequent downstroke until the end of the next upstroke; and (4) wbc 2, from the next upstroke until the end of its following downstroke (see Figures 7A, 8A).

Whereas head side-slip, resulting from a saccade, positively correlated with subsequent aerodynamic roll/pitch (ARP) body rotations that change the direction of aerodynamic force, head offset preceded subsequent proportional aerodynamic yaw (AY) body rotations that reorient the body about the aerodynamic force, but do not affect flight trajectory (Figures 7A,B). Saccade magnitude, however, did not linearly predict the magnitude of subsequent body rotations (Table S1). Associations of head side-slip and head offset with aerodynamic body rotations were of opposite sign and occurred at different subsequently timed wingbeat cycles. Head side-slip was positively associated with ARP over the wingbeat cycle (wbc 1) immediately following a saccade (Figures 7A,B) but negatively correlated with AY; whereas, head offset was positively associated with AY, but phase delayed over the second full wingbeat cycle (wbc 2) following the saccade (Figures 7A,C). Neither estimate of sensory input (head side-slip or head offset) predicted 3D body rotations, according to the linear regression models (Figures 7B,C). Notably, directional changes in horizontal head velocity (i.e., turning), which occurred over the subsequent 1.5 wingbeat cycles following a head saccade, were also strongly predicted by head side-slip (Figure 8; Table S1).

These statistical correlations for head side-slip with respect to ARP and AY, and for head offset with respect to AY, over subsequent wingbeat cycles (wbc 1 and 2) following a saccade (S) were robust: both regressions for individual subjects and least squares regression models corrected for turning direction and individual effects resulted in significant and consistent trends.

## Discussion

Throughout low-speed aerial turns pigeons stabilize their head in all three angular dimensions, despite continuous and independent oscillations of their body (Figures 4A,B). For these 90° level turns, translational fluctuations in head speed are horizontally directed and their peaks occur in-phase with the wingbeat cycle near the ventral downstroke to upstroke wing transition (Figure 3). Angular head saccades occur in nearly two-thirds of turning wingbeats (Figure 4), allowing for punctuated head stabilization periods during which pigeons greatly reduce head rotations and rotational optic flow (Videos S1,S2). Angular head saccades are also nearly horizontal, being directed into the turn and away from the bird's instantaneous flight trajectory. Saccade magnitude correlates with saccade timing and duration: larger saccades occur earlier in the wingbeat cycle, reach higher peak speeds and last slightly longer (Figure 5; Table S1).

The amount of head side-slip induced immediately after a saccade predicts body rotations that underlie flight trajectory changes (Ros et al., 2011) over the wingbeat cycle (wbc 1) immediately following the head saccade, as well as the resulting change in flight trajectory over the subsequent wingbeat cycle (wbc 2; Figures 7, 8). Conversely, head offset induced by neck bending immediately after a saccade correlates with changes in body orientation about the average direction of aerodynamic force (Ros et al., 2011) over the second wingbeat cycle following the saccade. Head offset thus predicts body rotations that realign the pigeon's body with the new flight trajectory, but which do not affect the flight trajectory itself (Figure 7).

The predominantly horizontal fluctuations in head speed likely serve a visual function. These translational head movement patterns during low-speed turns are similar to the head bobbing observed in pigeons prior to landing and reminiscent of the head bobbing observed during walking in many bird species, both of which are thought to provide visual cues for landing and object localization (Davies and Green, 1988; Green, 1998). Recently, we found similar head speed fluctuations associated with vertical and horizontal obstacle negotiation flight in pigeons (Ros et al., 2017) Consequently, peaks in translational head speed during turning flight (Figure 3) may also serve to improve parallax-based perception of speed and depth by increasing translational optic flow (Dunlap and Mowrer, 1930; Nakayama, 1985; Koenderink, 1986; Davies and Green, 1988; Zeil et al., 2008; Eckmeier et al., 2013). In addition, troughs in head speed (Figure 3) may function to reduce motion blur, facilitate detection of independently moving objects and, possibly, dishabituate ganglion cells involved in motion sensing (Frost and DiFranco, 1976; Nakayama, 1985; Necker, 2007; Frost, 2009). Furthermore, the consistent phase of head speed fluctuations (Figure 3) and angular saccades (Figure 4) with respect to the wingbeat cycle suggest that head motions and gaze changes are likely coupled to the central pattern generator network driving the flapping wings (Grillner, 2006). Although, this coupling must be loose since angular head saccades do not occur every wingbeat cycle (Figure 4D), and translational head saccades are absent during take-off flight (Davies and Green, 1988).

Importantly, the pigeon's head stabilization strategy isolates its head from body motions (Figures 3C,4A,B; see Videler et al., 1983; Bilo et al., 1985; Warrick et al., 2002; Eckmeier et al., 2008; Kress et al., 2015; Ros et al., 2017). As a result, both translational and angular head and body movements are uncorrelated, demonstrating reduced mechanical coupling effects between the head and body (Figures 3C,4A,B). Further, the transmission of strong, impulsive aerodynamic and inertial flight forces from the wings to the body suggests that head stabilization is actively mediated through cervical muscles controlled by optomotor and vestibulocollic reflexes (Gioanni and Sansonetti, 1999). The greater variability of head speed relative to the body compared to absolute head speed likely reflects active corrections of head position by cervical muscles that are needed to compensate for body motions during turning flight.

One advantage of fixating gaze through head orientation, as most birds do (Land, 1999), rather than by optical nystagmus of the eyes with respect to the head, as in humans and many other animals (Land, 1999), is that a stationary head may enable cervical sensors to provide information about the orientation of the body relative to the surroundings. Fast and robust control could be achieved by using cervical feedback as a single control input to steering muscles, as suggested by Groebbels (1929), effectively integrating visual and vestibular information. Alternatively, cervical feedback could be used to transform steering directions relative to the head into steering directions relative to the body. Such a head-to-body coordinate system transformation is necessary because external perception occurs within the head (eyes and vestibular systems), whereas steering motor output occurs within the body (wings and tail; see Krapp, 2010). Therefore, controlling head motion to stabilize the bird's visual field may not only facilitate extraction of important motion cues, but also offer a mechanism for controlling body and wing movements.

Our results show that potential offsets in visual feedback are primarily determined by head saccades during turning flight in pigeons (Figure 4). Because head saccades are consistently directed into the turn and away from the flight trajectory, the saccades generally lead to increases in head side-slip and, therefore, likely left:right optic flow asymmetry. Although saccades do not consistently start from zero head side-slip and not all of head rotation necessarily results in head side-slip, the large majority of head side-slip is determined by saccadic movement of the head. The pattern of angular gaze stabilization observed here is similar to that observed in pigeons held fixed while stimulated with an artificial visual surround under simulated flight conditions (Gioanni and Sansonetti, 1999), as well as in freely flying blowflies (Schilstra and van Hateren, 1998). Whereas pigeons mediate angular gaze stabilization largely through head movements, blowflies additionally use their whole body to stabilize gaze.

Whereas head side-slip predicts subsequent aerodynamic roll/pitch motions of the pigeon, head offset predicts subsequent aerodynamic yaw; yet neither correlates with full 3D body rotations according to the linear regression models (Figure 7). Based on our analysis of the potential sensory inputs that guide turning flight in pigeons, these key relationships indicate that flight trajectory and flight orientation are individually, yet concurrently, controlled through visual and cervical afferent inputs, respectively. However, it is unclear whether the delay between head side-slip immediately following a head saccade and the corresponding aerodynamic roll/pitch body rotations that occur in the next downstroke may be controlled through a dedicated visuomotor pathway. Recordings of *in vivo* muscle activation referenced to wing stroke timing (Ros et al., 2017) show that the onset of pectoralis muscle activation powering the downstroke occurs 23 ± 9 ms after the end of a saccade, when head side-slip is maximal. Temporal delays involved in optic flow processing via the AOS in birds at present, however, are unknown (Frost et al., 1990; Arends and Zeigler, 1991; Eckmeier et al., 2013). It is possible that, upon receiving parallel turning commands, the head may simply respond faster than the body, which is much heavier and slower to respond to aerodynamic forces that also require longer time periods to develop (Bilo, 1994). However, the absence of a correlation between saccade magnitude and subsequent body rotations (Table S1) makes the existence of such parallel turning commands less likely. Nevertheless, additional study of visual control of maneuvering flight in birds is needed to confirm a causal link between optic flow asymmetries and flight muscle control. Optic flow feature processing occurs in both the nBOR and the LM within the AOS. Both of these avian brain structures have extremely large receptive fields and integrate optic flow from the left and right eyes (e.g., McKenna and Wallman, 1981; Morgan and Frost, 1981). Therefore, these nuclei are the most likely candidates to process contralateral optic flow asymmetries. Notably, fruit flies similarly avoid optic flow foci of expansion appearing to originate from the flight direction, possibly to restore optic flow balance between the two eyes (Tammero et al., 2004).

Importantly, head side-slip also correlates with turning (i.e., changes in flight trajectory) over the wingbeat cycle following ARP body rotations that change the direction of aerodynamic force production (Figure 8). This temporal delay between the timing of aerodynamic roll/pitch rotations and of actual changes in flight trajectory indicates that pigeons must contend with a mechanical delay between executing sensorimotor commands that subsequently achieve a change in flight trajectory.

The correlation of head offset with subsequent aerodynamic yaw body rotations similarly suggests that head offset may control flight re-orientation of the bird's body after it has changed its flight trajectory (Figure 7). Based on our results, and consistent with past work on vestibulo-collic control of head position (Friedman, 1975; Frost, 1978; Gioanni and Sansonetti, 1999), it seems clear that visual feedback likely dominates as a sensory cue to cervical muscles controlling head orientation relative to the body. Thus, in-flight head stabilization may couple visual cues that guide changes in flight trajectory through aerodynamic roll/pitch with afferent cervical feedback that subsequently guides body re-orientation through aerodynamic yaw (Ros et al., 2011). The possible role of cervical afferents in controlling flight orientation is consistent with wing muscle activity in response to neck bending in pigeons under simulated flight conditions (Bilo and Bilo, 1978), and with the alignment response between the body and head associated with the vestibulo-collic reflex, as illustrated by the existence of a characteristic flight pose (Erichsen et al., 1989; Gioanni and Sansonetti, 1999). Diptera possess mechanoreceptors that sense their head angle such as the prosternal organ (Hengstenberg et al., 1986). Such interoceptive afferent feedback is similarly used to stabilize the head to improve vision, as well as to realign the body with the head and the vertical axis (Hengstenberg, 1984; Kern et al., 2006; Taylor and Krapp, 2007; Duistermars et al., 2012).

Our kinematic analysis of head, body and wing motions, in combination with prior work that has demonstrated limited eye movement relative to head movement underlying gaze stabilization in pigeons (Gioanni, 1988; Gioanni and Sansonetti, 1999; Land, 1999), indicates that pigeons rely heavily on visual information to guide their flight trajectory and possibly adjust their body orientation, based on head deviations relative to their body during turning flight. Future work is needed to test the predictions that emerge from these kinematic patterns and the sensorimotor control mechanisms they suggest. Nevertheless, our results strongly indicate that visual and proprioceptive cues are used as steering inputs for turning flight in birds, as has been observed for flying insects. This suggests the possibility of widespread flight control principles that can inspire design of robust controllers for human-engineered autonomous aerial vehicles.

## Author contributions

IR and AB: designed and performed research; IR: analyzed data; IR and AB: wrote the paper; AB: provided funding.

### Conflict of interest statement

The authors declare that the research was conducted in the absence of any commercial or financial relationships that could be construed as a potential conflict of interest.

## Supplementary material

Video S1 Aerial turn of a pigeon. A pigeon negotiating the 90° level turn, with markers on the torso, wings, and head.

Video S2 Angular head saccades. Four synchronized camera views, slowed down eight-fold, of a pigeon negotiating the 90° level turn. Each view is artificially centered on the head, illustrating angular head stabilization alternated with brief head saccades.
