# Supplementary material for: Pigeons (C. livia) Follow Their Head during Turning Flight: Head Stabilization Underlies the Visual Control of Flight
Source: Front Neurosci. 2017 Dec 1;11:655. doi: 10.3389/fnins.2017.00655 (PMC5717024; doi:10.3389/fnins.2017.00655)
Supplement: Supplementary file 3 [file Table1.pdf]

Table S1. Statistics for the Least Squares Linear Regression Models, corrected for effects of individual and turning direction (related figures indicated)

		adjusted R^2	Error DoF	F Ratio	p
Wingbeat phase vs					
	**Saccade amplitude**	0.49	26	29	**<.0001**
Saccade amplitude vs					
	**Saccade horizontal amplitude**	0.99	26	2661	**<.0001**
	**Saccade duration**	0.46	26	22	**<.0001**
(Fig. 5)	**Peak saccade speed**	0.83	26	139	**<.0001**
Head side-slip vs body rotations					
	3D_1	0.00	43	1	0.413
	3D_2	0.16	43	10	0.004
	3D_3	0.11	43	5	0.031
	3D_4	0.09	38	6	0.021
	ARP_1	0.00	43	2	0.210
	**ARP_2**	0.40	43	29	**<.0001**
	ARP_3	0.11	43	5	0.031
	ARP_4	0.20	38	12	0.002
	AY_1	0.09	43	6	0.021
	**AY_2**	0.25	43	16	**0.0003**
	AY_3	0.09	43	4	0.058
(Fig. 7)	AY_4	0.10	38	4	0.043
Head offset vs body rotations					
	3D_1	0.27	43	9	0.004
	3D_2	0.00	43	0	0.910
	3D_3	0.00	43	0	0.780
	3D_4	0.00	38	0	0.531
	ARP_1	0.22	43	7	0.015
	ARP_2	0.00	43	1	0.298
	ARP_3	0.00	43	2	0.210
	ARP_4	0.10	38	5	0.033
	AY_1	0.02	43	3	0.110
	AY_2	0.10	43	6	0.022
	**AY_3**	0.26	43	16	**0.0003**
(Fig. 7)	**AY_4**	0.55	38	46	**<.0001**
Head side-slip vs					
	Head velocity redirection_1	0.00	43	2	0.170
	Head velocity redirection_2	0.05	43	5	0.029
	**Head velocity redirection_3**	0.32	43	24	**<.0001**
(Fig. 8)	**Head velocity redirection_4**	0.53	38	47	**<.0001**
Saccade magnitude vs body rotations					
	3D_1	0.08	26	3	0.083
	3D_2	0.23	26	0	0.980
	3D_3	0.08	26	1	0.280
	3D_4	-0.10	25	1	0.347
	ARP_1	0.00	26	2	0.234
	ARP_2	0.02	26	0	0.719
	ARP_3	0.06	26	1	0.313
	ARP_4	-0.01	25	1	0.345
	AY_1	0.13	26	1	0.254
	AY_2	-0.02	26	0	0.745
	AY_3	0.04	26	2	0.194
	AY_4	0.17	25	0	0.550

On occasion a 4th complete wingbeat cycle defined as 'wbc 2' occurred later in the turn outside the calibrated kinematics volume, resulting in lower error DoF.
